# A comparing the strength of taekwondo turning and side kicks in relation to athletes’ gender, body weight and height

**DOI:** 10.1038/s41598-025-04785-9

**Published:** 2025-06-04

**Authors:** Tomasz Góra, Jacek Wąsik, Dariusz Mosler, Dorota Ortenburger, Mariusz Kuberski

**Affiliations:** Institute of Physical Culture Sciences, Jan Dlugosz University, Częstochowa, Poland

**Keywords:** Athletic performance, Motor skills, Taekwon-do, Gender differences, Biomechanics of martial arts, Biophysics, Anatomy

## Abstract

The aim of this study was to assess turning and side kick strength in the context of gender and lateralisation. One hundred and ninety-seven kicks performed by nine elite International Taekwon-do Federation athletes were analysed: four males and five females. To record the force of the impact, a strain gauge platform padded with a training disc was used as a target to protect the participants from direct impact on a force plate mounted on a stable structure. On average, the side kick achieves higher strength than the turning kick (*p* < 0.001). Significant elements differentiating kick strength values are gender and type of kick. The results of our study indicate that gender and kick type, as well as body weight and height, have a significant influence on kick pressure force. The study revealed significant differences in strength values between side kicks and turning kicks, with side kicks generally showing greater strength. On average, this pattern remained consistent for all genders. There was a significant relationship between gender, body mass and height and the kick and its strength. On average, greater kick pressure force was achieved by males, but some females, after specialized training, are able to achieve greater values of this dynamic indicator compared to male athletes. Although side kicks show higher strength values, the turning kick dominates taekwon-do competitions. This suggests that factors beyond strength alone, such as speed and control, influence the choice of technique in the context of competition.

## Introduction

Taekwon-do is martial art, with unique forms and self-defense techniques, which is expressed by unique physical, mental, and technical abilities of practitioners^1–3^. Among the many elements defining effectiveness in martial arts, the strength of strikes plays a crucial role in combat efficiency^4–6^. A high level of advancement in taekwon-do is often associated with the ability to generate substantial force during kicks^7,8^. Notably, the skillful use of body mass significantly affects the biomechanics of kicks^9–11^.

The turning kick and the side kick are two fundamental and most frequently used techniques used in taekwon-do^12–14^. Both techniques are effective for attacking an opponent from various directions and play a crucial role in sporting competitions. The turning kick is one of the most characteristic and spectacular elements of taekwon-do^7,15^. It is performed along the body’s axis in a circular motion, involving bending the knee and then extending it to strike with part of the foot. During the turning kick, the foot makes an arc in the air, approaching the opponent from the side. The turning kick can be executed at various heights, including the opponent’s head, torso, or legs. The side kick is characterized by a lateral position of the body and foot relative to the opponent, making it difficult to block or evade^16^. In a side kick foot travels in linear motion from the middle line of the body towards opponent. The primary difference between these two techniques is the direction of the strike and movement trajectory, showcasing its opposite nature and structure of movement. Turning kicks and side kicks show significant differences in execution patterns the former involves a rotational trajectory, while the latter follows a linear thrusting motion, resulting in different force generation mechanisms. Gender differences in sports, particularly in technical execution and force output, are significant due to inherent physiological and biomechanical variations between males and females. These differences can influence performance outcomes and training approaches, making them an important research topic. Physiological Differences: Men generally have larger muscle mass, greater skeletal-muscle mass, and higher maximal oxygen uptake, which contribute to differences in strength, speed, and power output compared to women^17,18^. These physiological traits are influenced by sex hormones, particularly testosterone, which is significantly higher in males^19^. Differences in muscle characteristics, such as fiber type and contractility, as well as neuromuscular regulation, contribute to variations in maximal force production between sexes^18,20^. For instance, males tend to have greater peak gluteus maximus force, while females may have higher forces in other gluteal muscles during running^18^. Gender differences can also vary by sport and position. For example, in field hockey, male players show a more velocity-oriented force-velocity profile, whereas female players may display a more force-oriented profile^20^ Additionally, sports requiring upper-body power show larger performance gaps between genders^21^.

Research into gender differences in sports is crucial for understanding how physiological and biomechanical factors influence performance. These insights can guide tailored training programs and improve performance outcomes while minimizing injury risks. Understanding these differences also helps in creating equitable opportunities and standards in sports.

However, the phenomenon of gender differences in martial arts is not well studied in the literature. Only one previous study, explores differences in kinematic variables on an example of front kick^22^. Researchers did not explore gender as an factor and focuses more on differences between i.e. styles of martial arts^23^. In contrast, phenomenon of laterality is consequently studied, indicating dominant side outperforming non-dominant in various settings and tasks^24^. However, in group of taekwon-do athletes, some researchers reports that there are no differences between dominant and non-dominant side while performing turning kick^25,26^. However, for this specific tasks, influence on generated force is yet to be known.

The effects of body weight and height on kicking force in martial arts are significant factors that influence performance. Understanding these effects can help athletes and coaches optimize training and improve competitive outcomes. There is a positive correlation between body mass and kicking force. Studies have shown that both novice and experienced martial artists can generate higher impact forces by effectively utilizing their body mass during kicks. This relationship is particularly evident in techniques like the front kick and side kick, where effective mass plays a crucial role in maximizing force output^27–29^. There is little research on the effect of body height on martial arts performance. It has been suggested that body height has no direct effect on martial arts performance, as factors such as weight management, strength and conditioning, and specific training programs have a greater impact on success^30^.

The strength of kicks in taekwondo is a crucial element of training that significantly influences effectiveness in combat^28^. However, achieving it requires not only strength training but also excellent technique and an understanding of the biomechanics of movement.

Therefore, the aim of this study was to investigate the differences in force output between side kicks and turning kicks, while evaluating the impact of gender, body weight, body height, and laterality (preferred vs. non-preferred leg) among elite Taekwon-do athletes. This study also aims to determine whether these factors significantly influence kick pressure force, and how these relationships manifest in both male and female practitioners.

The practical significance lies in refining athlete training strategies, as understanding which technique generates greater force under specific somatic and biomechanical conditions can guide individualized conditioning programs and tactical decisions in sparring or self-defence scenarios.

We hypothesize that (1) side kicks generate significantly higher pressure force than turning kicks, and (2) kick force is significantly influenced by gender, height, weight, and leg preference.

## Materials and methods

### Participants

One hundred and ninety-seven kicks performed by nine elite ITF (International Taekwon-do Federation) athletes were analysed: four males (aged 28.5 ± 6.5 years, body mass 77.5 ± 6.1 kg, height 180.0 ± 1.4 cm) and five females (aged 27.0 ± 4.8 years, body mass 64.2 ± 5.8 kg, height 163.0 ± 6.5 cm). To be included in the study group, participants had to meet the following criteria: be at least 18 years old, possess a minimum of a black belt, have at least eight years of training experience, actively compete in national-level sports for at least four years, have won at least one Polish championship title, and be free from injuries. Testing conditions were standardized for all participants, with assessments conducted in the morning at a consistent room temperature. In the laboratory where the taekwon-do athletes were examined, artificial lighting from fluorescent lamps prevailed. The light was cool, with a distinctly white hue characteristic of this type of source. It was evenly dispersed throughout the room, eliminating shadows and providing good visibility for precise measurements and observations. The examination took place on puzzle mats, which were evenly laid out on the floor. These mats had a characteristic non-slip texture and provided adequate cushioning, which was particularly important for the safety of the athletes while performing kicks.

Participants were fully informed about the testing procedures and voluntarily provided their consent to participate in data collection. The Human Subjects Research Committee of Jan Dlugosz University reviewed and approved the ethical protocol (KE-O/4/2022). All participants were free of injuries, thoroughly briefed on the testing procedures, and willingly agreed to take part in the study.

The research was conducted in strict accordance with the relevant guidelines and regulations outlined in the Declaration of Helsinki, ensuring adherence to ethical principles for medical research involving human subjects. This included obtaining written informed consent from all participants, safeguarding the confidentiality and anonymity of collected data, and minimizing any potential risks associated with the study.

### Protocol

**Fig. 1:**
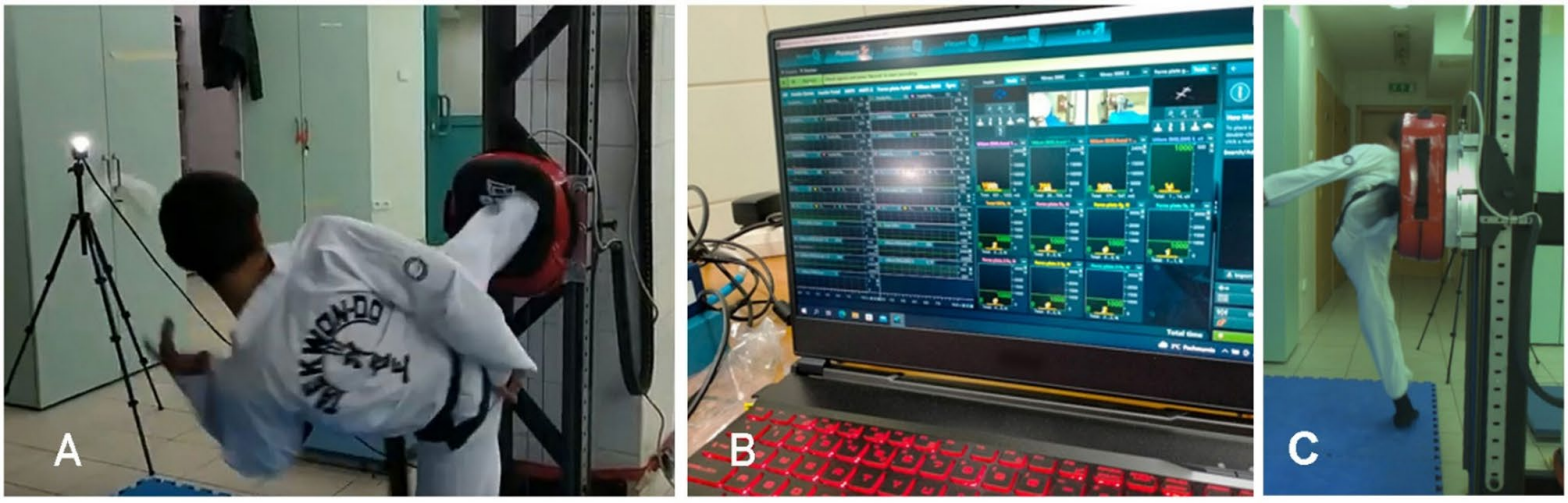
Example photographs from the study: (**A**) Measurement of the side kick, (**B**) View of the software interface on the computer, (**C**) Measurement of the turning kick.

To measure impact force, a force plate was utilized as the target, shielded by a training pad to prevent direct contact from participants. The force plate, an AMTI model MC12-2 K from the 2000 series (Watertown, MA, USA) with a measurement frequency of 580 Hz, measured 305 × 406 × 79 mm and was securely fastened to a stable structure. This setup was synchronized temporally and spatially with the Noraxon system (MR3 3.18) to ensure precise data capture. Data collection occurred at the Center for Human Movement Analysis, Jan Dlugosz University, Czestochowa. Participants started with a self-directed 10-min warm-up. They then performed five right-leg side kicks (yop chagi) targeting the force plate from a sport stance. After a one-minute rest, they executed five right-leg turning kicks (dollyo chagi) towards the same target, also from a sport stance. This procedure was then repeated with the left leg for all participants. In a few cases, the registration of four or six kicks occurred due to human performance error or an error in the registration system. The athletes adjusted the height of the target to their preference, staying within the allowable striking zones outlined in ITF taekwon-do competitions. They positioned it at the level of their solar plexus to optimize the impact of the kick for maximum power. Throughout the test, participants were instructed to use maximum effort to generate the highest possible impact force, with no time constraints on the execution of the kicks. A total of 197 trials were recorded, comprising 109 trials for female and 88 trials for male. Sample photos of measurements are shown in Fig. 1.

### Data processing and statistical analysis

Data collection involved recording five strokes for each technique per participant. Initially, the data was exported from the measurement software in Excel *.slk format and then converted to *.xlsx format for easier processing. Maximum force values were determined using a Python (3.12) script using the scipy library and the findpeaks function to identify peak forces. These maximum force values were then organised and collated in Microsoft Excel 365 for statistical analysis. A total of 180 peak values were extracted, calculated from the number of participants (9), number of trials (5), types of techniques (2) and sites used (2). The collected data was averaged.

Once the data were prepared, they were exported to Statistica 13 software (TIBCO). Basic descriptive statistics were calculated and the normality of the data distribution was assessed using the Shapiro-Wilk test (W = 0.912; *p* < 0.001). Since the normality condition was not met, the Wilcoxon test was performed to determine the significance of the differences in the strength of selected kicks and in between the right and left legs. In addition, beta coefficients were calculated using a multivariate regression model to examine any relationship between pressure force, gender, type of kick and leg (left or right), weight and body height. We used the partial and semi-partial regression coefficient is used to express the specific portion of variance explained by regression model. Heteroscedasticity phenomena were assessed using a visual method. The level of statistical significance was set at *p* < 0.05.

## Results

Tables 1 and 2 presents the medians force values of the studied kicks, categorized by gender, type of kick, and leg (left and right). Significant differences were observed between the right and left legs in the average force values for the selected kicks. For women, the turning kick showed a right leg force of 1440.18 N and a left leg force of 1347.43 N (Z = 1.371; *p* = 0.170); for the side kick, the right leg force was 1900.32 N and the left leg force was 1651.62 N (Z = 0.168; *p* = 0.866). For men, the turning kick resulted in a right leg force of 2793.90 N and a left leg force of 2602.35 N (Z = 1.639; *p* = 0.101); for the side kick, the right leg force was 4911.97 N and the left leg force was 4828.22 N (Z = 1.639; *p* = 0.101). As expected, men exhibited higher force values in their kicks compared to women (Z = 3.757; *p* < 0.001).

**Fig. 2:**
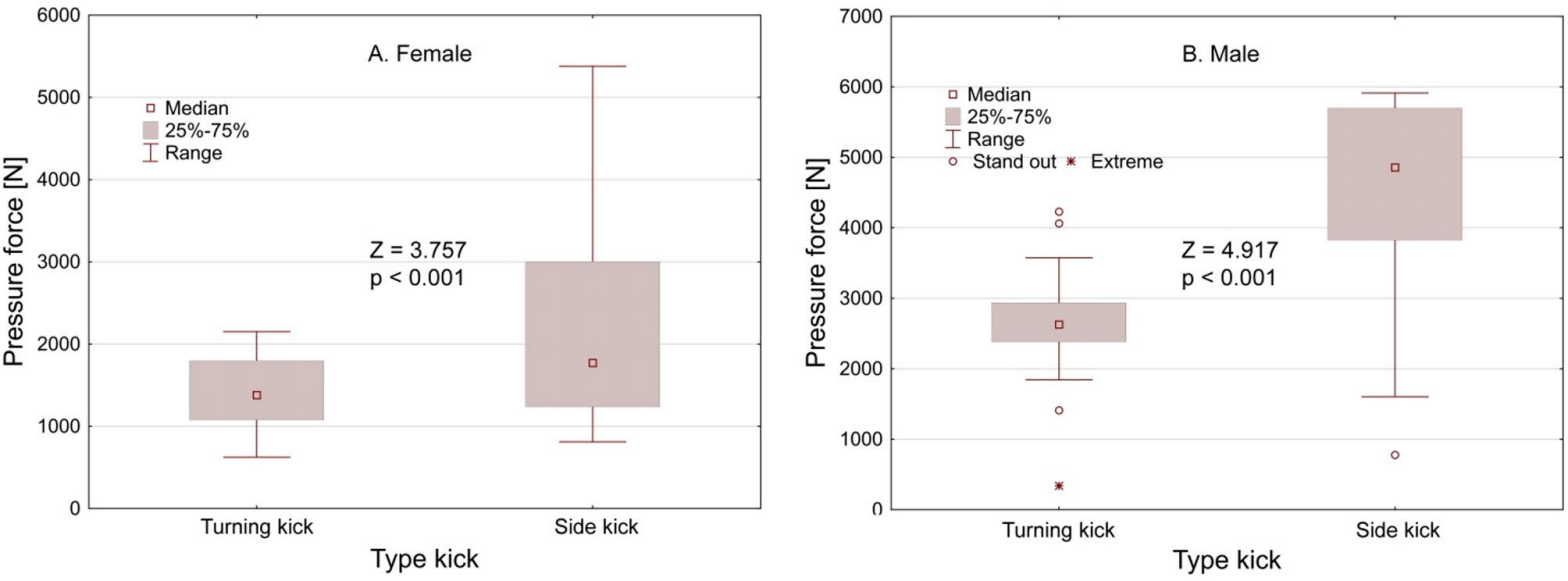
Graphic representation of the pressure force for turning kick and side kicks by gender.

Figure 2 graphically illustrates the median force of the turning and side kicks by gender, indicating that the side kick generally achieves higher force values than the turning kick (females: Z = 3.757; *p* < 0.001; males: Z = 4.917, *p* < 0.001).

Table 3 contains the results based on the multiple regression model. A significant relationship was identified between pressure force and the gender of the players represented (beta = 0.939; *p* < 0.001) among the recorded kicks. Additionally, a moderate relationship was observed between kick type (beta = 0.437; *p* < 0.001) and body height (beta = – 0.487; *p* < 0.001) and a discernible correlation with body weight (beta = 0.143; *p* = 0.03). The similarity between R² (0.623) and Adjusted R² (0.62) in this case suggests that most predictors in the model contribute meaningfully to explaining the variability in kick pressure force. This balance between R² and Adjusted R² reinforces the validity of the regression model for explaining kick pressure force, ensuring both accuracy and generalizability.

**Fig. 3:**
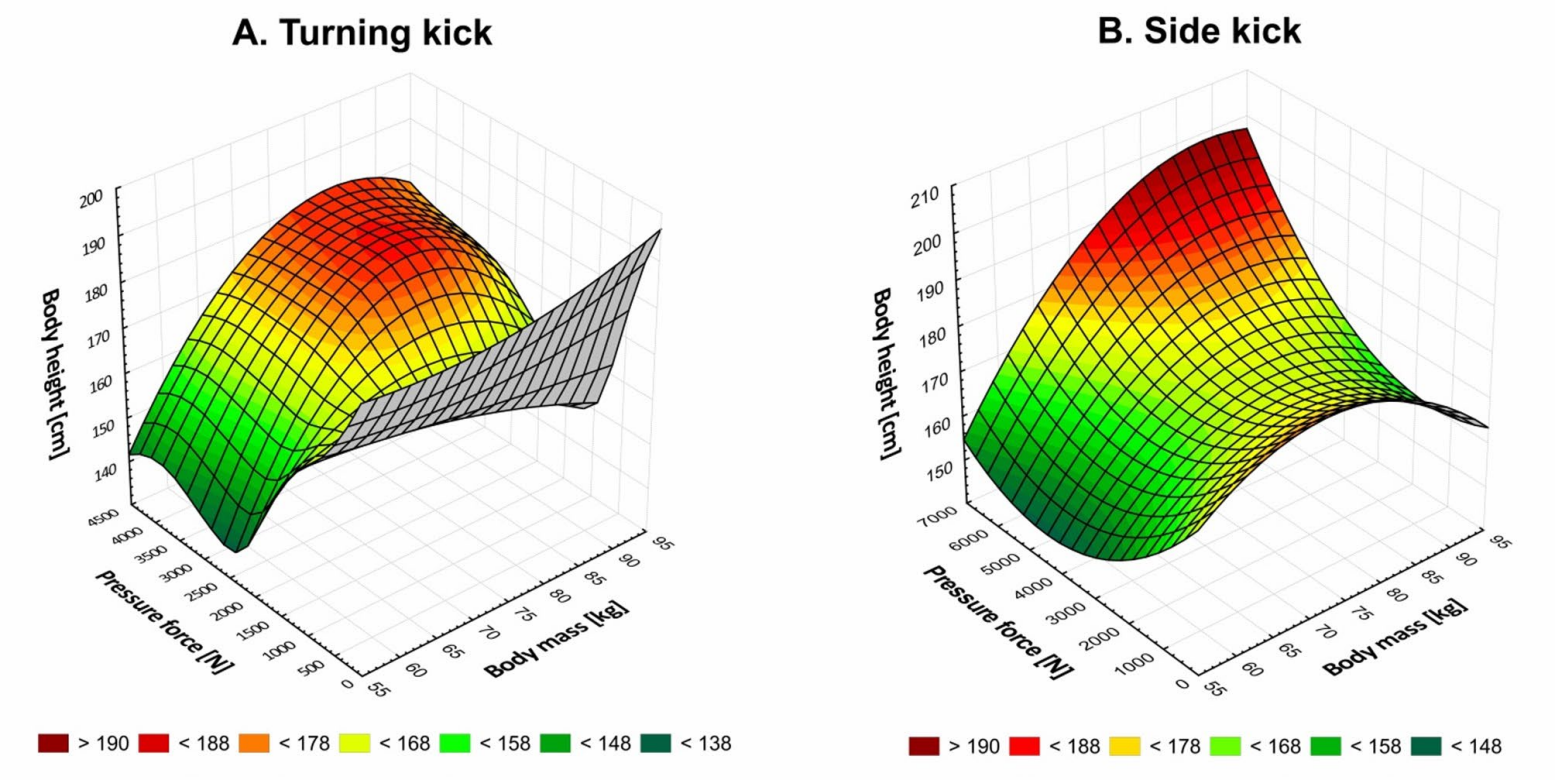
Relationship between kick pressure force and athlete’s body mass and height.

Figure 3 presents a graphical representation of the relationship between kick pressure force, athlete’s body weight, and height. It can be observed that the relationship between the selected somatic characteristics is different for the type of kick. In the case of the side kick, the observed trend shows that the pressure force exerted by the side kick increases with increasing body weight and height. However, the relationship between these indicators for the turning kick is different and less clear.

**Table 1:** Median pressure force values during the execution of selected kicks by female athletes according to kick type and leg.

Kick Type	Leg	Q1	Median	Q3	Min	Max	Statistic	Statistic
Turning kick	Right	1120.74	1440.18	1711.04	706.26	2030.76	Z = 1.371; *p* = 0.170	Z = 3.757; *p* < 0.001^*^
	Left	998.82	1347.43	1811.37	624.82	2150.55		
Side kick	Right	1258.15	1900.32	2939.71	811.70	3841.44	Z = 0.168; *p* = 0.866	
	Left	1193.03	1651.62	3236.90	821.26	5379.02		

^*^Statistically significant, Q1 - first quartile, Q3 - third quartile, Min – minimum, Max – maximum.

**Table 2:** Median pressure force values during the execution of selected kicks by male athletes according to kick type and leg.

Kick Type	Leg	Q1	Median	Q3	Min	Max	Statistic	Statistic
Turning kick	Right	2452.51	2793.90	3054.86	1857.55	4227.96	Z = 1.639; *p* = 0.101	Z = 4.917; *p* < 0.001^*^
	Left	2312.28	2602.35	2733.02	338.096	3249.13		
Side kick	Right	3902.71	4911.97	5820.81	776.567	5912.73	Z = 1.639; *p* = 0.101	
	Left	3213.87	4828.22	5525.12	1602.49	5842.95		

^*^Statistically significant, Q1 - first quartile, Q3 - third quartile, Min – minimum, Max – maximum.

**Table 3:** Multiple regression model of state kick pressure force level in the context predictor variables: *R* = 0.79, R^2^ = 0.623, adjusted R^2^ = 0.62, F(5.190) = 64.035 *p* < 0.001, standard error of estimate: 877.56. ^*^ statistically significant.

Predictors	BETA	Standard error	t (190)	*p*
Gender	0.939	0.107	8.754	< 0.001^*^
Kick type	0.437	0.044	9.845	< 0.001^*^
Leg	-0.027	0.044	-0.618	0.536
Body mass	0.143	0.065	2.178	0.030^*^
Body height	-0.487	0.089	-5.44	< 0.001^*^

While the analysis presented detailed comparisons between legs, the initial research question emphasized gender-related differences in kicking strength. To address this, regression analysis revealed a statistically significant impact of gender on kick force (β = 0.939, *p* < 0.001). On average, male participants produced higher force, yet individual female athletes, following specialized training, occasionally exceeded male outputs. Thus, the observed variability supports gender-influenced performance trends.

## Discussion

The conducted research reveals that the force value of the turning kick in taekwon-do athletes ranges from 624 to 2150 N for women and 1409 to 4227 N for men; for the side kick, it ranges from 811 to 5379 N for women and 2023 to 5912 N for men. This is consistent with the findings of other researchers^31^. The recorded minimum and maximum values show significant variance, despite all participants being from an advanced and experienced group of athletes. It is hypothesized that these differences may stem from individual specializations and preferences. The ITF taekwon-do sporting competition includes various events, one of which is the ‘power test’ that involves breaking statically positioned boards. Our measurement approach was very similar to this event. Therefore, under these conditions, ‘specialists’ might have achieved better results.

Our measurements reveal that the side kick achieves, on average, higher force values than the turning kick. Nonetheless, research results show that athletes use turning kick most often in sport fighting^32^. Thus, other aspects must determine the choice of technical arsenal in this type of competition. Studies report that the turning kick achieves a higher velocity than the side kick^33^ and, as reported by other authors, the speed of movement does not necessarily affect the power of the kick^15^. Consequently, the high speed of movement and the ease of execution of the kick in a situation where the situation on the mat (or ring) is in full control allows us to obtain the set targets. However, in a situation where we do not have to fear the reaction of the opponent and the value of the force generated will be the main priority the use of the side kick may be a better solution. In a self-defence situation, we may be faced with the dilemma of whether to strike once, but as strongly as possible according to the right of one opportunity^34^. Such a dilemma can be crucial in what are termed borderline situations, where the manner in which an act is taken can mean the difference between life and death. For example, destroying a closed door in a burning building in order to save a child.

In our analyses, significant differences were not between the right and left leg in the mean force values when performing both the turning kick and the side kick. This is consistent with other studies showing that ITF taekwon-do athletes do not observe significant differences between maximum velocities and movement times when executing selected right and left leg kicks^35^. Such symmetry is important for the successful execution of kicking techniques. This may be due to training habits that build muscle symmetry. Taekwon-do athletes often focus on training both limbs equally, as many techniques require the use of both the right and left legs. Long-term, targeted training may lead to balanced muscle development and functional symmetry between limbs. Although we reference general principles of motor learning and compensation, further taekwon-do-specific neuromuscular studies are needed.

The results of our study indicated that gender, as well as body weight and height, have a significant impact on kicking strength. The observed trend indicates that the pressure force exerted by the side kick increases with increasing body weight and height. However, the relationship between these indicators at the turning kick is less clear, which calls for further research. Achieving increased kicking power can be facilitated by strengthening leg muscles and trunk stabilizers. This can be achieved by implementing plyometric exercises, refining movement patterns and optimizing limb synchronization to improve kick biomechanics. Additionally, the use of elastic resistance has been shown to promote improvements in movement speed^33^. An increase in side kick strength can be promoted by training that builds body mass. Turning kick, on the other hand, can be improved by refining technique and flexibility. The current visualizations (e.g., Fig. 2) present some challenges in clarity, especially the overlapping data and compressed axis range. Future revisions will aim to improve figure clarity by adopting clearer data visualization methods such as separate scatterplots or regression overlays.

Of course, it is not surprising that we record, on average, a higher strength of kicks performed by men. However, our research shows that some women, after specialised training, are able to achieve higher values for this dynamic indicator than male athletes. Thus, anthropometric differences do not necessarily determine the effectiveness of a punch. This suggests that technical proficiency—such as optimized hip rotation, timing, or limb coordination—may play a greater role than purely anthropometric traits. In other sports, long-term training has been shown to minimize anthropometric disadvantages. While this principle may apply to taekwon-do as well, more discipline-specific longitudinal studies are needed to confirm its effects^36,37^. Although we reference the concept of effective mass, this study did not include motion capture or 3D kinematic analysis. Therefore, any inferences about movement technique are speculative and based on indirect evidence from force output.

Another revealing factor in the regression process carried out is the moderating effect of body mass on kick strength. It can therefore be assumed that the use of greater mass will be an element that increases the recorded force on the dynamometric platform during the execution of this type of movement. Obviously, a person with more mass naturally puts more of his body weight into the impact. However, research suggests that one possibility for an increase in mass during a kick is an appropriate movement technique resulting in an increase in so-called effective mass^9,11,38^. Thus, a person with a lower total body mass, using the right movement patterns, is able to gain more or similar effective mass than an objectively heavier athlete. Current research shows that the side kick achieves greater effective mass than the turning kick, but lower foot acceleration values^38^.

Limitations of the study include the small sample size and the collection of data under laboratory conditions, which may not fully reflect real-world conditions. Future research should include a larger and more diverse group of athletes at different skill levels and age groups. Similar analyses on other strokes and the effect of specific training programs on kicking strength, as well as psychological factors such as self-confidence on effectiveness, seem interesting.

It is hoped that this work will partially fill the gap regarding martial arts biomechanics research, although it considers only a small part of the problem. We believe that this study expands the knowledge of the biomechanical relationships of martial arts performance and may serve as a reference for other researchers and can pave the way for further research.

## Limitations

This study has several limitations that should be acknowledged. Firstly, the small sample size, although composed of elite-level athletes, restricts the generalizability of the findings to broader populations, including lower-skilled or younger practitioners. The repeated-measures design partially compensates for this by increasing internal consistency, but further validation on larger and more diverse cohorts is necessary.

Secondly, the study focused exclusively on impact force as the primary performance metric, without incorporating electromyographic analysis or kinematic variables such as joint angles, velocity, or timing. As such, muscle activation patterns and neuromuscular strategies underlying the differences in force output remain unexplored.

Third, the differences in anthropometric characteristics between male and female participants (especially height and weight) were accounted for statistically but not normalized directly in the raw force values. Future studies should consider normalization methods or controlled matching to refine inter-gender comparisons.

Finally, the study was conducted under laboratory conditions with a fixed target height and controlled environment, which may not fully replicate the dynamic context of real combat scenarios. Further research should include in-situ measurements during sparring or competition to improve ecological validity.

## Conclusion and practical implications

The present study demonstrated that side kicks produce significantly greater impact force than turning kicks, regardless of gender. While male athletes generally exhibited higher kick forces, selected female athletes with specialized training reached comparable or superior results, emphasizing the importance of technique and conditioning over purely anthropometric factors.

Regression analysis confirmed that kick type, gender, body mass, and body height all contribute significantly to variations in pressure force, with leg dominance (left vs. right) showing no significant effect among this high-level athlete cohort. This finding highlights the symmetrical development promoted by elite-level taekwon-do training.

From a practical standpoint, these results suggest that: Training strategies should be tailored to optimize each athlete’s biomechanical strengths — athletes with higher body mass may benefit more from techniques that capitalize on effective mass (e.g., side kick), while lighter athletes might prioritize speed and precision (e.g., turning kick). Female athletes can achieve competitive striking forces with focused strength and technical training, reinforcing the case for individualized programming rather than gender-based expectations. Technical coaches might consider emphasizing the development of both techniques to ensure tactical versatility — the turning kick, while less forceful, may offer strategic advantages in speed and angular approach. Conditioning protocols should aim at maximizing effective mass and core stability, which play a crucial role in force transmission during kicking.

Overall, the study contributes to the growing field of martial arts biomechanics by providing insights into how force output in taekwon-do kicking is shaped by individual factors, and by offering evidence-based directions for sport-specific training programs.

## Data Availability

The datasets used and analysed during the current study available from the corresponding author on reasonable request.
